# Anti-Inflammatory and Antioxidant Effects of (6*S*,9*R*)-Vomifoliol from *Gaultheria procumbens* L.: In Vitro and Ex Vivo Study in Human Immune Cell Models

**DOI:** 10.3390/ijms26041571

**Published:** 2025-02-13

**Authors:** Piotr Michel, Anna Wajs-Bonikowska, Anna Magiera, Agnieszka Wosiak, Ewa Balcerczak, Monika Ewa Czerwińska, Monika Anna Olszewska

**Affiliations:** 1 Department of Pharmacognosy, Faculty of Pharmacy, Medical University of Lodz, Muszyńskiego 1, 90-151 Lodz, Poland; anna.magiera@umed.lodz.pl (A.M.); monika.olszewska@umed.lodz.pl (M.A.O.); 2 Institute of Natural Products and Cosmetics, Faculty of Biotechnology and Food Sciences, Lodz University of Technology, Stefanowskiego 2/22, 90-537 Lodz, Poland; anna.wajs-bonikowska@p.lodz.pl; 3 Laboratory of Molecular Diagnostics and Pharmacogenomics, Department of Pharmaceutical Biochemistry and Molecular Diagnostics, Medical University of Lodz, Muszyńskiego 1, 90-151 Lodz, Poland; agnieszka.wosiak@umed.lodz.pl (A.W.); ewa.balcerczak@umed.lodz.pl (E.B.); 4 Department of Biochemistry and Pharmacogenomics, Faculty of Pharmacy, Medical University of Warsaw, Banacha 1, 02-097 Warsaw, Poland; monika.czerwinska@wum.edu.pl; 5 Centre for Preclinical Research, Medical University of Warsaw, Banacha 1B, 02-097 Warsaw, Poland

**Keywords:** vomifoliol, blumenol A, *Gaultheria procumbens*, LOX, neutrophils, PBMCs, *NF-κB* gene expression

## Abstract

(6*S*,9*R*)-vomifoliol (VO) is a natural norisoprenoid of the megastigmane type derived from *Gaultheria procumbens*, an aromatic, evergreen shrub whose leaves, fruits, and aerial parts are used in traditional phytotherapy to treat oxidative stress and inflammation-related disorders. The plant is known as a rich source of essential oil and polyphenols. However, the levels of other constituents of *G. procumbens*, including VO, have yet to be explored. There is also a knowledge gap in the pharmacological potential of VO in the context of inflammation. Therefore, the present study aimed to investigate the accumulation of VO in leaves, stems, and fruits of *G. procumbens* and to determine its antioxidant and anti-inflammatory effects in non-cellular in vitro and cell-based models of human immune cells ex vivo. The GC-FID-MS (gas chromatography coupled with flame ionisation detector and mass spectrometer) analysis revealed the leaves as the richest source of VO (0.36 mg/g dw of the plant material) compared to other *G. procumbens* organs. In non-cellular activity tests, VO showed comparable to positive control anti-inflammatory activity against lipoxygenase, with significantly weaker impact on hyaluronidase and cyclooxygenase-2, and no effect on cyclooxygenase-1 isozyme. VO at 5–75 μM revealed a significant and dose-dependent ability to reduce the reactive oxygen species (ROS) level, downregulate the release of pro-inflammatory cytokines [tumour necrosis factor-α (TNF-α), interleukin-8 (IL-8), IL-6, and IL-1β] and tissue-remodelling enzymes (elastase-2, metalloproteinase-9), and up-regulate the secretion of anti-inflammatory cytokine IL-10 in bacterial lipopolysaccharide (LPS)- and *N*-formyl-L-methionyl-L-leucyl-L-phenylalanine (*f*MLP)-stimulated human neutrophils and peripheral blood mononuclear cells (PBMCs) ex vivo. Furthermore, a significant reduction in *IL-6*, lipoxygenase (*LOX*), nuclear factor κ-light-chain-enhancer of activated B cells 1 (*NF-κB1*), and *NF-κB2* gene expression in LPS-stimulated peripheral blood lymphocytes was demonstrated by real-time PCR. The cellular safety of VO at 5–75 μM was confirmed by flow cytometry, with the viability of neutrophils and PBMCs after incubation with VO at 93.8–98.4%. The results encourage further studies of VO as a promising non-cytotoxic natural anti-inflammatory agent and support the use of leaves of *G. procumbens* in the adjuvant treatment of oxidative stress and inflammation-related diseases of affluence.

## 1. Introduction

**Figure 1:**
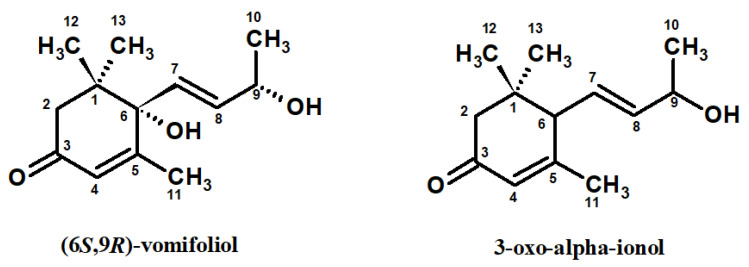
Structure of (6*S*,9*R*)-vomifoliol (VO) isolated from *G. procumbens* leaves [4] and its precursor 3-oxo-α-ionol.

(6*S*,9*R*)-6-hydroxy-3-oxo-α-ionol [(6*S*,9*R*)-vomifoliol; blumenol A; VO] is a natural lipophilic norisoprenoid, a derivative of 3-oxo-α-ionol (Figure 1). VO belongs to the megastigmane-type norisoprenoids with a C_13_ skeleton derived from the oxidative degradation of β-carotenoids [1]. Megastigmane derivatives and their glycosides are widely distributed in the plant kingdom and reported to possess wide-ranging biological properties, making them active constituents of several plants and promising candidates for drug development [1]. As a representative of megastigmanes, VO occurs in the form of four stereoisomers, i.e., (6*S*,9*S*) = corchoionol C, (6*S*,9*R*) = blumenol A, (6*R*,9*S*), and (6*R*,9*R*) [2]. Although the most natural VO isomers have a (6*S*)-configuration, stereoisomers possessing a (6*R*)-configuration and their glycosides were also isolated from plant sources [3].

The accumulated results of non-cellular in vitro and antimicrobial research suggest that VO has anti-inflammatory [5,6], antioxidant [6,7,8,9,10,11], antidiabetic [9], anti-acetylcholinesterase [12,13,14], antibutyrylcholinesterase [14], antityrosinase [6], antiviral [7], antibacterial [6,13,15], antifungal [13], and antiparasitic [12] potential. VO also presented anti-inflammatory effects in cell-based models. It significantly inhibited the nitric oxide (NO) production in bacterial lipopolysaccharide (LPS)-activated murine microglia cells N9 [16] and the RAW 264.7 macrophages [17,18], reduced the production of interleukin-2 (IL-2) by phorbol 12-myristate-13-acetate/ionomycin-stimulated Jurkat cells [19], and downregulated the IL-6 and tumour necrosis factor-α (TNF-α) secretion and NO production in LPS-stimulated RAW264.7 macrophages [7]. VO exhibited also neuroprotective effects against amyloid-beta (Aβ1-42)-induced cytotoxicity in neuroblastoma SH-SY5Y cells [20]. The literature thus suggests that anti-inflammatory and antioxidant activity is the most promising for VO. This aligns with our previous studies, which led to the isolation of VO from *G. procumbens*, a traditional anti-inflammatory herbal medicine [4]. However, there are still no reports on the influence of VO on human immune cells, which play primary roles in inducing and resolving inflammation. 

Neutrophils, the most abundant blood morphotic elements engaged in the immunity, first respond to pro-inflammatory factors. Under activated conditions, they secrete a plethora of pro-inflammatory cytokines, including interleukin-1β (IL-1β), IL-8, and TNF-α; many enzymes involved in inflammation, like cyclooxygenase-2 (COX-2), lipoxygenases, tissue-remodelling matrix metalloproteinase-9 (MMP-9) and human elastase-2 (ELA-2); and reactive oxygen species (ROS) [21]. Human peripheral blood mononuclear cells (PBMCs) are a variety of specialised immune cells, such as monocytes/macrophages and lymphocytes, produced in the bone marrow from hematopoietic stem cells and coordinating the immune system. Activated PBMCs also produce a wide array of cytokines, such as TNF-α and IL-6, in non-specific inflammatory responses. Besides pro-inflammatory ones, PBMCs also release anti-inflammatory cytokines, e.g., interleukin-10 (IL-10), which might inhibit the synthesis of pro-inflammatory cytokines and resolve inflammation [22]. On the other hand, the accumulation of pro-inflammatory factors might aggravate the pathogenesis of inflammation-related diseases. Hence, modulation of the immune cell functions could be an effective strategy for treating several disorders mediated by oxidative stress and inflammation.

The ability to modulate the pro-inflammatory and prooxidant functions of human neutrophils is well-documented for numerous herbal medicines used for treating inflammation-related disorders, including *G. procumbens*, a source plant for VO [23,24,25,26]. As indicated by traditional medicine, rheumatoid arthritis, influenza, the common cold, fever, and pain of various aetiology are therapeutic targets for *G. procumbens* [23]. Previously, leaves, fruits and aerial parts of this ericaceous, evergreen shrub have been found to accumulate significant amounts of active compounds, primarily volatiles and polyphenols, like salicylates, flavonoids, and proanthocyanidins [23,24,25,26]. However, the levels of VO in *G. procumbens* have yet to be investigated.

Therefore, the aim of the present study was (a) to characterise the leaves, fruits, and stems of *G. procumbens* as a source of VO; (b) to evaluate the anti-inflammatory activity of VO towards four pro-inflammatory enzymes in vitro; (c) to analyse the antioxidant and anti-inflammatory potential of VO in human immune cells ex vivo, including neutrophils and PBMCs; and (d) to determine the effect of VO on the gene expression of pro-inflammatory factors in PBMCs. The GC-FID-MS (gas chromatography coupled with flame ionisation detector and mass spectrometer) method was used to determine the content of VO in *G. procumbens* leaf, fruit, and stem chloroform extracts. The anti-inflammatory activity of VO in non-cellular models was examined towards important pro-inflammatory enzymes, including COX-2 and COX-1 isoforms. Moreover, the release of a series of pro-oxidant, pro-inflammatory, and anti-inflammatory factors (ROS, IL-1β, IL-6, IL-8, TNF-α, MMP-9, ELA-2, and IL-10) was examined in neutrophils and PBMCs. The relative expression ratio (R) of the *IL-6*, lipoxygenase (*LOX*), nuclear factor κ-light-chain-enhancer of activated B cells 1 (*NF-κB1*), and *NF-κB2* genes in PBMCs was determined by the real-time PCR and calculated by the double-delta method. Furthermore, the cellular safety of VO was verified by flow cytometry.

## 2. Results

### 2.1. VO Content in G. procumbens Extracts

The chloroform dry extracts of leaves (L.CHE), fruits (F.CHE), and stems (S.CHE) of *G. procumbens* were prepared by direct extraction of the plant material in a Soxhlet apparatus, according to the previously described scheme [4]. The extracts varied in extraction yield, as shown in Table 1, with the highest value observed for L.CHE. The content of VO in all three extracts was determined by the GC-FID-MS analysis with the internal standard and trimethylsilyl derivatisation (Table 1). The representative GC chromatograms of L.CHE, F.CHE, and S.CHE are in Figure S1. The results displayed L.CHE as the richest source of VO [0.36 mg/g dry weight (dw) of the leaves] among the tested extracts. VO content in the F.CHE was below the detection limit.

### 2.2. Effect on Pro-Inflammatory Enzymes in Non-Cellular Models

The anti-inflammatory activity of VO in non-cellular models was tested towards four pro-inflammatory enzymes (Table 2). The concentration-dependent inhibitory activity of VO against LOX was comparable to that of the natural anti-inflammatory agent nordihydroguaiaretic acid (NDGA). In the case of hyaluronidase (HYAL) and COX-2, the VO effect was significantly weaker than that of the positive controls: NDGA, acetylsalicylic acid (ASA) and celecoxib (CX). The tested compound did not inhibit COX-1 activity over the entire concentration range (1.54% of enzyme activity inhibition at 9.00 mM).

### 2.3. Effect on Neutrophils and PBMC Viability

**Figure 2:**
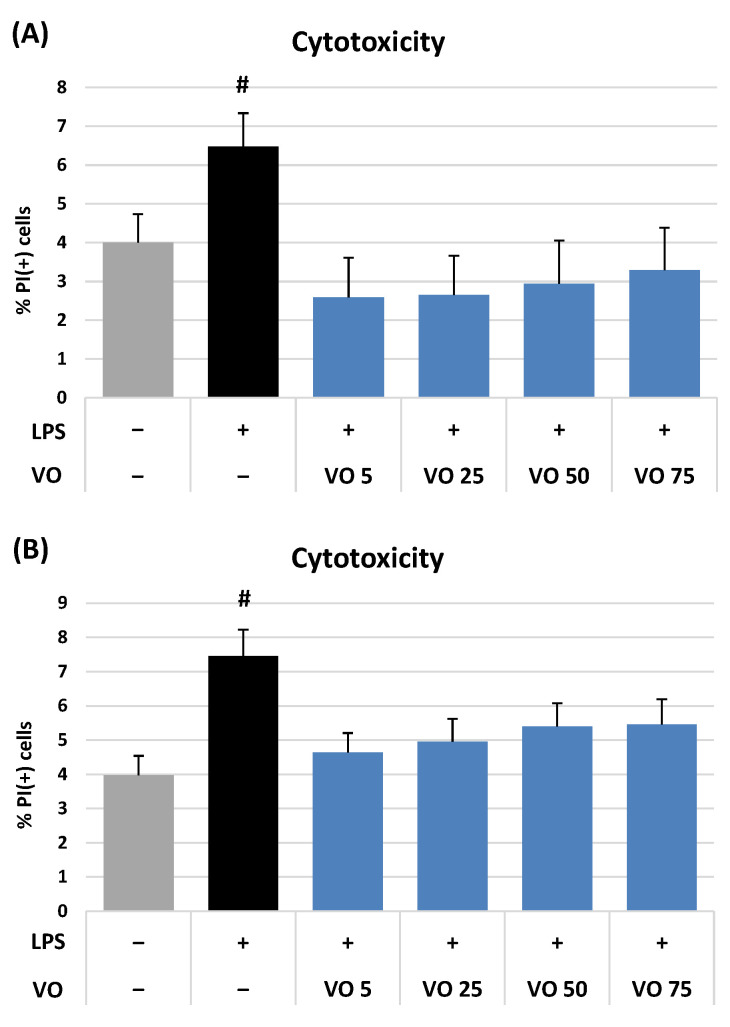
Effect of (6*S*,9*R*)-vomifoliol (VO) at 5–75 µM on viability (membrane integrity) of (**A**) neutrophils and (**B**) peripheral blood mononuclear cells (PBMCs), as indicated by propidium iodide positive PI(+) cells. Data expressed as means ± SD of three independent experiments performed with cells isolated from five independent donors. Statistical significance: # *p* < 0.05 compared to the non-stimulated control.

The potential cytotoxicity of VO at 5–75 μM was examined by propidium iodide staining with flow cytometry. The viability of neutrophils and PBMCs after incubation with VO was 93.8–98.4% (Figure 2) and did not differ significantly (*p* > 0.05) from that of LPS-stimulated control (91.8–94.4%) and non-stimulated cells (95.3–96.7%). Therefore, VO might be considered non-cytotoxic to the tested human immune cells. 

### 2.4. Effect on ROS Production by Neutrophils

**Figure 3:**
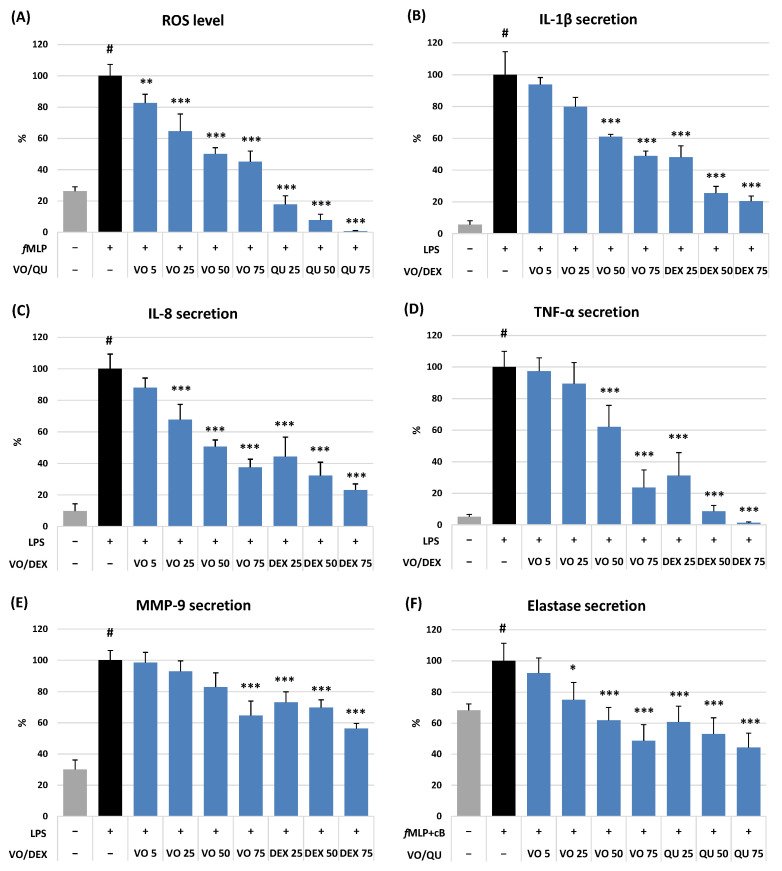
Effect of (6*S*,9*R*)-vomifoliol (VO) at 5–75 µM on (**A**) reactive oxygen species (ROS) level and secretion of (**B**) interleukin-1β (IL-1β), (**C**) IL-8, (**D**) tumour necrosis factor-α (TNF-α), (**E**) matrix metalloproteinase-9 (MMP-9), and (**F**) elastase-2 (ELA-2) by stimulated human neutrophils. Data expressed as means ± SD of three independent experiments performed with cells isolated from five independent donors. Statistical significance: # *p* < 0.001 compared to the non-stimulated control untreated with the compound; * *p* < 0.05, ** *p* < 0.01, *** *p* < 0.001 decreased compared to the stimulated control untreated with the compound. Positive controls: quercetin (QU) and dexamethasone (DEX) were tested at 25–75 µM.

The cellular antioxidant activity of VO was evaluated by measuring its impact on ROS production by human neutrophils stimulated with *N*-formyl-L-methionyl-L-leucyl-L-phenylalanine (*f*MLP). VO revealed a significant (*p* < 0.01) and dose-dependent antioxidant effect within the whole concentration range of 5–75 μM (Figure 3A). For instance, at the highest tested level, VO downregulated the ROS release by 54.8% compared to the *f*MLP-stimulated control.

### 2.5. Effect on the Release of Pro- and Anti-Inflammatory Cytokines and Enzymes

**Figure 4:**
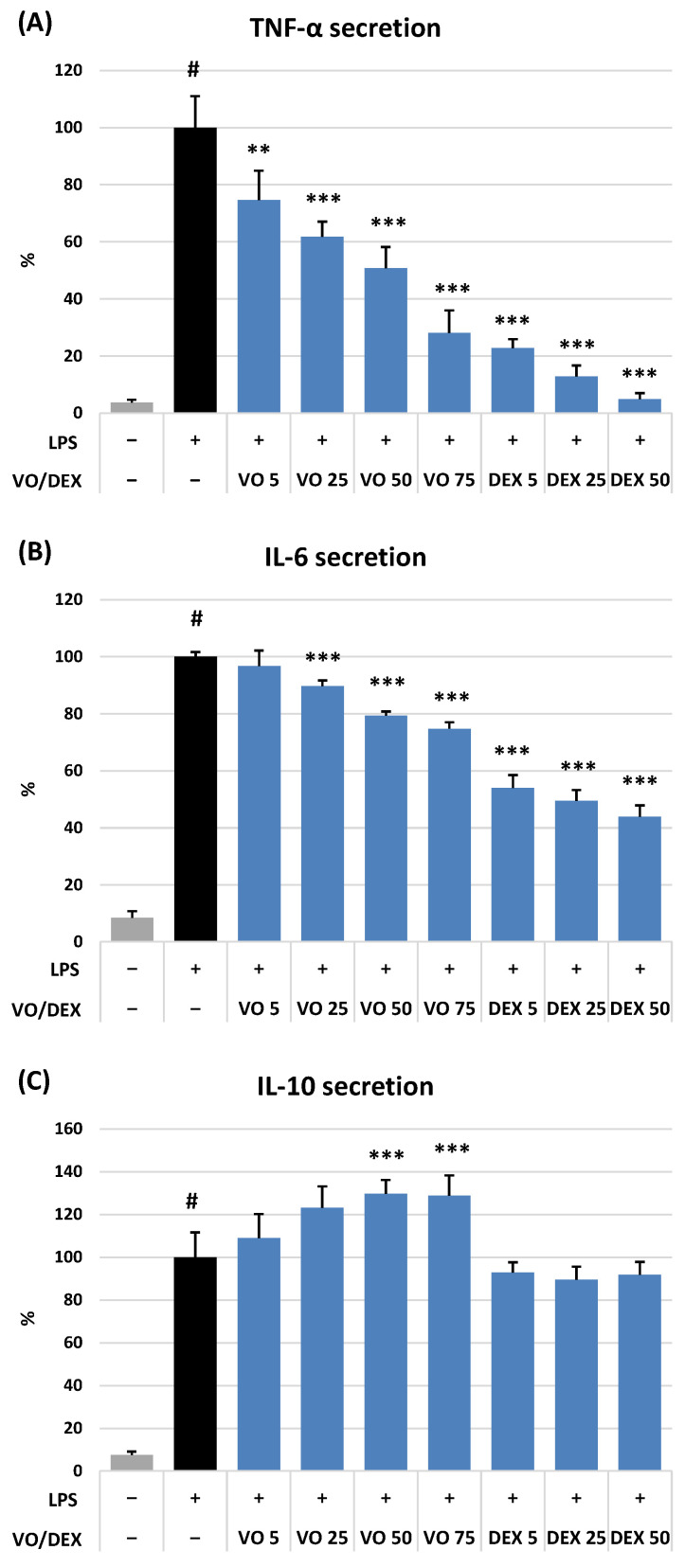
Effect of (6*S*,9*R*)-vomifoliol (VO) at 5–75 µM on the secretion of (**A**) interleukin-6 (IL-6), (**B**) IL-10, and (**C**) tumour necrosis factor-α (TNF-α) by stimulated human peripheral blood mononuclear cells (PBMCs). Data expressed as means ± SD of three independent experiments performed with cells isolated from five independent donors. Statistical significance: # *p* < 0.001 compared to the non-stimulated control untreated with the compound; ** *p* < 0.01, *** *p* < 0.001 decreased compared to the stimulated control untreated with the compound. Dexamethasone (DEX) was tested as a positive control at 5–50 µM.

As illustrated in Figure 3B–F and Figure 4A–C, VO demonstrated a concentration-dependent ability to modulate the release of pro-inflammatory (IL-6, IL-8, IL-1β, and TNF-α) and anti-inflammatory (IL-10) cytokines and tissue remodelling enzymes (ELA-2 and MMP-9) produced by neutrophils and PBMCs after stimulation by LPS or *f*MLP with cytochalasin B, depending on the test. The observed effects were the most potent for TNF-α, both in neutrophils (Figure 3D) and PBMCs (Figure 4A). In the presence of VO at 75 μM, the release of this cytokine decreased to 23.6% and 28.0% (*p* < 0.001) for neutrophils and PBMCs, respectively, and did not differ significantly from the effect of dexamethasone (DEX) at 25 μM (*p* > 0.05).

The effect of VO on the rest of the pro-inflammatory cytokines was less pronounced but still statistically significant at 25–75 μM (IL-8 and IL-6) and 50–75 μM (IL-1β). Exposure of stimulated neutrophils and PBMCs to VO at 75 µM resulted in significant inhibition of IL-8, IL-1β, and IL-6 release down to 37.5%, 48.9%, and 74.8% compared to the stimulated control (expressed as 100%; *p* < 0.001), respectively. As shown in Figure 4C, the VO also significantly affected the anti-inflammatory IL-10 secretion at higher concentrations (50–75 μM). The stimulatory effect reached a maximum (29% increase) at 75 μM.

In the case of pro-inflammatory enzymes released by stimulated neutrophils, VO was a more potent inhibitor of ELA-2 than MMP-9 secretion (Figure 3E,F). For instance, VO at 75 μM downregulated the secretion of ELA-2 and MMP-9 by 51.3% and 35.4%, respectively, compared to the stimulated control (*p* < 0.001). Moreover, in the case of ELA-2, the VO effect at the highest tested concentration was similar to that of quercetin (QU) at 75 μM (*p* > 0.05).

DEX, a potent anti-inflammatory drug (5–75 μM), significantly (*p* < 0.001) reduced the release of all pro-inflammatory cytokines and enzymes in both cellular models (Figure 3B–E and Figure 4A–C) but did not influence (*p* > 0.05) the IL-10 production in PBMCs (Figure 4C). At the same time, QU, a powerful antioxidant agent (5–75 μM), noticeably (*p* < 0.001) diminished the levels of ROS (Figure 3A) and ELA-2 (Figure 3F) released by activated neutrophils, compared to the stimulated control (*p* < 0.001).

### 2.6. Effect on the Gene Expression of Pro-Inflammatory Factors

**Figure 5:**
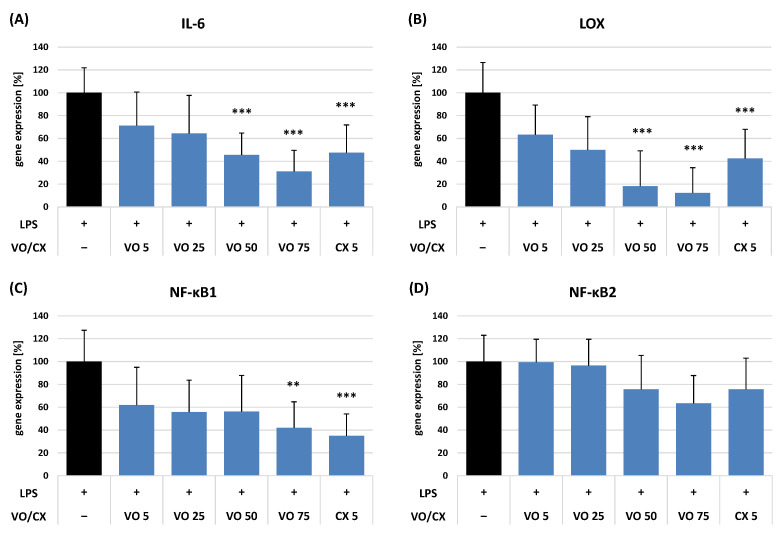
Effect of (6*S*,9*R*)-vomifoliol (VO) at 5–75 μM on the expression of (**A**) interleukin-6 (*IL-6*), (**B**) lipoxygenase (*LOX*), (**C**) nuclear factor κ-light-chain-enhancer of activated B cells 1 (*NF-κB1*), and (**D**) *NF-κB2* genes by bacterial lipopolysaccharide (LPS)-stimulated human peripheral blood mononuclear cells (PBMCs). The calculated level of gene expression was normalised relative to the reference glyceraldehyde 3-phosphate dehydrogenase (*GAPDH*) gene and using the results for unstimulated control untreated with the compound as a calibrator. Data expressed as means ± SD of three independent experiments performed with cells isolated from five independent donors. Statistical significance: ** *p* < 0.01, *** *p* < 0.001 compared to the stimulated control untreated with the compound. Celecoxib (CX) was tested as a positive control at 5 µM.

The mechanism of the anti-inflammatory activity of VO at 5–75 μM was evaluated by measuring its impact on the *IL-6*, *LOX*, *NF-κB1*, and *NF-κB2* gene expression in LPS-stimulated PBMCs by qRT-PCR, with the expression levels calculated using the double-delta method (Figure 5).

The observed effects were concentration-dependent and the strongest for the gene expression of *LOX* and *IL-6*, and to a lesser extent also for *NF-κB1* and *NF-κB2*. Incubation of the stimulated PBMCs with VO at 50 μM and 75 μM resulted in a significant reduction in the *LOX* gene expression down to 18.2% and 12.3% and *IL-6* gene expression down to 45.5% and 31.1%, respectively, compared to the LPS-treated control cells (*p* < 0.001). More importantly, the activity of VO was comparable to a reference CX at 5 μM. VO also downregulated the expression of the *NF-κB1* gene, but only at the highest tested concentration. In those conditions, the *NF-κB1* gene expression decreased to 42.0% (*p* < 0.01) compared to the LPS-stimulated cells, which meant a similar effect to that of CX at 5 μM (*p* > 0.05). The impact of VO on the *NF-κB2* gene expression was also concentration-dependent but statistically insignificant over the entire concentration range.

## 3. Discussion

The current study validated and ascertained the anti-inflammatory and antioxidant potential of (6*S*,9*R*)-vomifoliol (VO; Figure 1) in non-cellular in vitro tests and human immune cells ex vivo. VO used in this work for biological research was previously isolated from chloroform extract of *G. procumbens* leaves by chromatographic techniques as one of the active constituents of the plant [4]. *G. procumbens* is a small, aromatic, evergreen shrub whose leaves, fruits, and aerial parts are used in phytotherapy to treat oxidative stress and inflammation-related disorders, including rheumatoid arthritis, influenza, the common cold, and fever [23]. The plant is a rich source of volatiles and polyphenols [23]. However, there is no literature data on the levels of VO in *G. procumbens*.

Therefore, the present study started with verifying and comparing different organs of *G. procumbens* as potential sources of VO. For this purpose, the leaves, fruits, and stems of the plant were independently extracted with chloroform and analysed for the VO content by GC-FID-MS (Figure S1). The results indicated that only leaves with high extraction yield and an abundance of the target compound at 0.36 mg/g dw (Table 1) might serve as a natural source of VO, while in other organs, the VO content is at most negligible. To our knowledge, this is the first report on the accurate quantification of VO in *Gaultheria* sp.; previously, only a relative percentage level was reported for trimethylsilylated components of the *G. procumbens* leaf extract [4]. Moreover, most previous works on other plants also concerned only the isolation and identification of VO in plant materials or their volatile fractions using various chromatographic and spectroscopic techniques without actual quantitative measurements [27,28,29,30]. Few studies reported the VO content, e.g., in wines obtained from the Nerello Mascalese [31,32] and Tannat [33] red grape varieties, fresh red grapes of Corvina, Corvinone, and Rondinella varieties [34], and berry epicarp of the red grape variety Cesanese [35]. However, in all these products, the VO level did not exceed 2 μg/L of wine and 2.5 μg/g dw of the plant materials. Thus, *G. procumbens* leaves are a rich source of VO that may be efficiently recovered from the plant sample by simple, direct extraction with chloroform.

In the following research stage, the direct inhibition of pro-inflammatory enzymes responsible for activating signalling pathways of the inflammation process was tested as one of the possible mechanisms of anti-inflammatory effects of VO, previously confirmed for various *Gaultheria* extracts [24,25]. The first enzyme, LOX, regulates the inflammatory response by generating essential pro-inflammatory mediators, such as leukotrienes and lipoxins [36]. The second one, HYAL, breaks down hyaluronan—a fundamental component of the extracellular matrix that links protein filaments, collagen fibres, and connective tissue cells, thus contributing to the spread of inflammation [37]. The third group is formed by cyclooxygenases, chief enzymes converting arachidonic acid to prostanoid mediators of acute and chronic inflammation, including prostaglandins, prostacyclins, and thromboxanes. However, only COX-2, the inducible enzyme form, is purely pro-inflammatory among the two main COX isoenzymes. In contrast, COX-1, although involved in some inflammatory processes, is most of all constitutively expressed in numerous tissues and engaged in, among others, protecting the stomach mucosa and platelet aggregation [38]. In our study, comparative analysis of the inhibitory potential of VO towards four pro-inflammatory enzymes in vitro showed relevant impact on the LOX. VO presented comparable to the positive control anti-inflammatory activity against LOX, with a significantly weaker impact on HYAL and COX-2 and a lack of effects towards the COX-1 isoenzyme (Table 2). The ability to selectively inhibit COX-2 with no impact on COX-1 is a pharmacologically preferred feature of anti-inflammatory drugs, essential for preventing undesirable adverse treatment effects, such as gastric ulceration and decreased platelet aggregation, often observed for nonselective COX inhibitors [39]. Therefore, VO seemed a good candidate for further investigating its anti-inflammatory effects in cellular models.

The previous studies [5,6,7,8,10,11] and the present findings proved the prominent direct anti-inflammatory and antioxidant activity of VO in non-cellular in vitro assays. However, the indirect cellular mechanisms are more important in vivo [40]. For this reason, and because of the lack of data on the influence of VO on the inflammation-related functions of human immune cells, VO was next tested ex vivo in human neutrophils and PBMCs, isolated from blood plasma buffy coats.

One of the essential steps in biological research on natural compounds is the assessment of their cytotoxicity. Our findings suggest that VO, even at a wide range of 5–75 μM concentrations, did not reduce the viability of human neutrophils and PBMCs (Figure 2). The cellular biocompatibility of VO has been previously demonstrated for several normal cell lines, both animal and human. For instance, Tan et al. [20] showed that VO at 1–20 μM did not exhibit significant toxicity towards the SH-SY5Y neuroblastoma cells within 72 h of exposure. Similarly, Zhang and co-workers [19] showed no significant cytotoxicity of VO against murine splenocytes and Jurkat cells during 48 h incubation at 1–30 μM and 1–40 μM, respectively. On the other hand, there are also a few reports on the cytotoxic effects of VO on several cancer cell lines [17,41,42,43], suggesting the anticancer potential of the compound. In conjunction with all these data, our results confirm the compound as a non-cytotoxic candidate for further biological research on normal cells.

Oxidative stress and inflammation are deeply interconnected processes associated with the pathogenesis of chronic diseases. During the pathological stimulation, activated immune cells like neutrophils, PBMCs, and macrophages generate large amounts of ROS. This overproduction leads to lipid peroxidation, DNA damage, and protein oxidation, resulting in localised oxidative stress and tissue injury. The ensuing tissue injury triggers a cascade of events leading to the exaggerated inflammatory response and elevated release of ROS, pro-inflammatory cytokines, and vasoactive factors. Additionally, ROS act as signalling molecules that activate multiple pro-inflammatory pathways, transcription factors, and receptors, such as NF-κB or NOD-like receptor protein 3 (NLRP3) inflammasome, collectively amplifying the immune response. Therefore, it is crucial to interrupt this self-reinforcing cycle to combat chronic diseases associated with oxidative stress and inflammation effectively [44].

In the present study, we have observed that VO can significantly and in a dose-dependent manner downregulate the pro-oxidant and pro-inflammatory functions of stimulated human neutrophils and PBMCs (Figure 3 and Figure 4). The highest effectiveness of VO was observed towards the ROS production by neutrophils, the secretion of TNF-α, both in neutrophils and PBMCs, and the release of ELA-2 by activated neutrophils. Previously, VO was confirmed to inhibit the overproduction of TNF-α in LPS-stimulated murine RAW264.7 macrophages [7] but was not effective in IL-1β-induced SW982 synovial cells [45], which may suggest that the effects of VO are cell-specific. TNF-α, produced predominantly by activated macrophages and lymphocytes, is a powerful priming agonist of neutrophils and a pleiotropic master cytokine that orchestrates the inflammatory cascade and stimulates gene expression of numerous chemokines and enzymes, including COX and LOX. TNF-α also modulates the crucial step in the development of inflammation, which is the recruitment and adhesion of immune cells to vascular endothelium [46]. The downregulation of TNF-α has become a cornerstone in treating various inflammatory and autoimmune disorders, including psoriasis, rheumatoid arthritis, and ankylosing spondylitis [47]. ELA-2 is a tissue remodelling metalloproteinase that breaks down the extracellular matrix components, such as elastin and collagen, which can lead to increased permeability, massive release of inflammatory mediators, and exaggerated infiltration of immune cells to the site of inflammation, creating a feedback loop that exacerbates the process [48]. The reduction in ELA-2 activity holds significant promise as a therapeutic approach for managing some inflammation-related diseases, such as rheumatoid arthritis, inflammatory bowel disease, chronic obstructive pulmonary disease, and cystic fibrosis [49]. All these specific diseases responsive to anti-TNF-α and anti-ELA-2 treatment are thus potential targets for the therapeutic application of VO, providing a clear direction for future research.

In the last step of this work, the effect of VO on the expression of four genes encoding important pro-inflammatory factors, i.e., NF-κB1, NF-κB2, LOX, and IL-6, was verified for LPS-stimulated human PBMCs. NF-κB is a pivotal transcription factor controlling innate and adaptive immune responses through regulating over 200 genes involved in cell function and inflammation [50]. The proteins encoded by *NF-κB1* and *NF-κB2* genes are produced as precursor polypeptides, namely NF-κB1 p105 and NF-κB2 p100, which, after proteolytic degradation by the 26S proteasome, release the mature transcription factors NF-κB1 p50 and NF-κB2 p52, respectively [51]. These p50 and p52 proteins take a crucial part in three separate NF-κB activation pathways. The canonical and NF-κB1 p105 pathways trigger the release of p50/RelA heterodimers and p50/p50 homodimers, respectively, which, after nuclear translocation and gene transcription regulation, have an essential impact on immune responses, the development and spread of inflammation and cell survival promotion. On the other hand, the noncanonical or alternative NF-κB pathway requires the processing of NF-κB2 p100 to p52 protein, and it is essential for secondary lymphoid organogenesis, maturation of B-cells, and adaptive humoral immunity [52]. The *LOX* gene family encodes a group of enzymes known as lipoxygenases, which are involved in the biosynthesis of various lipid mediators, including leukotrienes and hydroxyeicosatetraenoic acids, which are critical players in the inflammatory response. LOX contributes to the inflammatory milieu by generating pro-inflammatory factors that promote synovial inflammation, joint destruction, bronchoconstriction, mucus production, and airway remodelling, all characteristic of several inflammatory diseases [53]. IL-6 is involved in immune responses, inflammation processes, and haematopoiesis, including neutrophil production in the bone marrow [54]. Moreover, IL-6 is an activator of the signal transducer and activator of transcription 3 (STAT3) factor, which interacts synergistically with the NF-κB signalling pathway and induces NF-κB hyper-activation called “IL-6 amplifier” followed by over-production of various pro-inflammatory cytokines and chemokines [55]. In our work, VO presented substantial and statistically significant inhibitory activity towards especially *LOX* and *IL-6*, but also *NF-κB1* gene expression (Figure 5). All of these factors are reported as targets for anti-inflammatory therapies against, e.g., asthma and allergic diseases, rheumatoid arthritis, inflammatory bowel disease, chronic obstructive pulmonary disease, psoriasis, and cytokine release syndrome [36,56,57]. Thus, inhibition of *NF-κB1*, *LOX*, and *IL-6* gene expression by VO represents an attractive approach for therapeutic intervention in inflammatory and autoimmune diseases.

VO, as a sesquiterpene-derived, lipophilic norisoprenoid, offers some additional benefits in the context of medicinal application. Sesquiterpenes are low-molecular-weight compounds rapidly absorbed after oral administration, presenting high oral bioavailability and quickly penetrating the skin when applied topically [58]. Chaves et al. [59] reported a good bioavailability of the sesquiterpene α-humulene in mice when applied orally, which remained detectable up to 12 h after administration. Dish et al. [60] demonstrated satisfactory absorption capacity for sesquiterpene petasin and its derivatives in tested intestinal segments, using an in situ rat model. Jürgens et al. [61] also showed high dermal absorption (over 97% penetration) after 48 h of *Arnicae* tincture sesquiterpene lactones of helenalin type into porcine and human skin cells ex vivo. Thus, good bioavailability could potentially characterise VO as a sesquiterpene derivative. This aspect, together with a significant reduction in the gene expression of pro-inflammatory factors, inhibition of pro-inflammatory enzyme activity, and modulation of pro-inflammatory and pro-oxidant responses of human immune cells ex vivo, might predispose this α-ionol derivative as a potential anti-inflammatory drug and a model compound for the design of new effective and bioaccessible therapeutic agents against several inflammatory diseases. However, more in-depth studies are required to evaluate the molecular mechanisms of the anti-inflammatory activity of VO in vitro and in vivo. The bioavailability and potential toxicity of VO, in addition to the levels of administration necessary to achieve health-promoting effects, are also aspects in need of further research.

## 4. Materials and Methods

### 4.1. Plant Material and VO Reference Standard

Leaves, stems, and fruits of *G. procumbens* L. were collected in October 2023 in the gardening centre of Ericaceae plants, Gospodarstwo Szkolkarskie Jan Cieplucha (54°44′ N, 19°18′ E), Konstantynow Lodzki (Poland), where the plants grew in an open area. The seed origin and authentication were reported previously [24]. The voucher specimens (KFG/HB/23001-GPRO-STEMS; KFG/HB/23001-GPRO-FRUITS; KFG/HB/23001-GPRO-LEAVES) were deposited in the Medicinal Plant Garden, Medical University of Lodz (Poland). The plant name has been checked with the WFO Plant List (2024) [62]. Samples of the plant material were air-dried at 35 °C, powdered with an electric grinder, and sieved through a ø 0.315 mm sieve.

VO was isolated previously with the HPLC purity of 99.3% from the leaves of *G. procumbens* by open-column and flash chromatography and identified by spectroscopic (MS/MS, NMR, UV, IR) and optical rotation experiments [4].

### 4.2. Preparation of Chloroform Extracts

The preparative sample (10 g) of the pulverised plant material was extracted independently in a Soxhlet apparatus with chloroform (1 L, 72 h) as described previously [4]. The obtained extracts were concentrated in vacuo to give leaf (L.CHE), fruit (F.CHE), and stem (S.CHE) chloroform dry extracts. The extraction yield was calculated per the plant material dry weight (dw).

### 4.3. Quantification of VO by GC-FID-MS Analysis

The analyses were performed on a Trace GC Ultra coupled with a DSQII mass spectrometer (Thermo Electron Corporation, Waltham, MA, USA). A simultaneous GC-FID-MS analysis was performed using an MS FID splitter (SGE Analytical Science, Milton Keynes, UK). The mass range was 33–550 amu, with an ion-source heating of 200 °C and an ionisation energy of 70 eV. Operating conditions: capillary column Rtx-1 MS (60 m × 0.25 mm i.d., film thickness 0.25 μm), temperature programme: 60–150 °C at 1 °C/min and to 300 °C (30 min) at 4 °C/min. Injector and detector temperatures were 280 °C and 300 °C, respectively. The carrier gas was helium (constant pressure: 300 kPa). 

The quantification of VO in the extracts was based on an internal standard method with heptadecane C_17_H_36_ (98.5% pure, Sigma Aldrich, St. Louis, MO, USA) used as an internal standard. The samples of the dry extracts (50 mg/mL) were dissolved in trichloromethane (99.8% pure, Chempur, Piekary Śląskie, Poland), and then 200 μL of standard solution (0.14 mg/mL) in heptane (analytical pure, Chempur, Piekary Śląskie, Poland) was added. Samples were derivatised with BSTFA + TMCS [*N*,*O*-bis(trimethylsilyl)trifluoroacetamide + trimethylchlorosilane; 200 μL] (analytical standard, Cerilliant Corporation, Round Rock, TX, USA) for 3 h in the dark. After this time, the samples were injected (1 μL) into the GC-MS system in triplicate.

### 4.4. Biological Activity Tests

#### 4.4.1. Evaluation of Anti-Inflammatory Activity in Non-Cellular Models

The ability of VO to inhibit LOX and HYAL was evaluated according to Matczak et al. [63], and the ELISA tests (detection range: 3.9–500 pg/mL) for COX-1 and COX-2 followed the manufacturer’s instructions (Cayman Chemical, Ann Arbour, MI, USA). The results were expressed as IC_50_ values. VO and positive controls were tested at the final concentrations of 0.025–0.9 mM, 0.025–1 mM, 0.25–9.00 mM, and 0.01–4.50 mM for the LOX, HYAL, COX-1 and COX-2 tests, respectively. NDGA, ASA, and CX were used as positive controls. A detailed description of the procedures is included in the Supplementary Materials. All reagents and standards were purchased from Sigma-Aldrich (St. Louis, MO, USA). All non-cellular anti-inflammatory tests were performed using 96-well plates and a microplate reader SPECTROstar Nano (BMG Labtech, Ortenberg, Germany).

#### 4.4.2. Isolation of Neutrophils and PBMCs from Human Buffy Coats

Neutrophils and PBMCs were isolated from human buffy coats obtained from five independent donors by dextran sedimentation, erythrocyte lysis, and centrifugation in a Ficoll-Hypaque gradient (PAA Laboratories, Pasching, Austria), as previously described [24,64]. After isolation, the cells were suspended in RPMI 1640 culture medium (BioWest, Nuaillé, France), (Ca^2+^)-free phosphate-buffered saline (PBS; Thermo Fisher Scientific, Waltham, MA, USA), or (Ca^2+^)-free Hanks’ balanced salt solution (HBSS; Sigma-Aldrich, St. Louis, MO, USA), depending on the test, and were stored at 4 °C until use. The buffy coats for this study were purchased from the Warsaw Regional Blood Centre (Poland). Peripheral venous blood for fractionation and preparation of buffy coats was collected in the centre from healthy male volunteers (ages 18–35 years). The donors were non-smokers, clinically recognised as healthy, and did not take any medications. This study complied with the principles of the Declaration of Helsinki. As it used a by-product material available on the market, it did not require the approval of the bioethics committee.

#### 4.4.3. Viability Assessment of Neutrophils and PBMCs

The potential cytotoxicity of VO was assessed by flow cytometry (BD FACSCalibur, BD Biosciences, San Jose, CA, USA) with propidium iodide (PI) staining and Triton X-100 solution as a positive control, according to Michel et al. [24] and Magiera et al. [64] for neutrophils and PBMCs, respectively. The final test concentrations of VO were 5–75 μM. The analyses were performed after 24 h and 48 h incubation for neutrophils and PBMCs, respectively. Cells treated with Triton X-100 solution (98.6% and 99.3% of PI(+) cells) were used as positive controls in the neutrophil and PBMC models, respectively. A detailed description of the procedure is included in the Supplementary Materials.

#### 4.4.4. Evaluation of ROS, IL-8, IL-1β, IL-6, IL-10, TNF-α, MMP-9, and ELA-2 Secretion by Human Immune Cells

All assays were performed as previously described [24,64]. Briefly, the ROS production by neutrophils stimulated by *N*-formyl-L-methionyl-L-leucyl-L-phenylalanine (*f*MLP) was determined using the luminol-amplified chemiluminescence assay. The release of MMP-9 by neutrophils stimulated by LPS was evaluated by the sandwich ELISA test (detection range: 31.2–2000 pg/mL) following the instructions (R&D Systems, Minneapolis, MN, USA). The *f*MLP+cytochalasin B-induced secretion of ELA-2 by the cells was measured using *N*-succinyl-alanine-alanine-valine-*p*-nitroanilide (SAAVNA) as a substrate. The release of pro- and anti-inflammatory cytokines (IL-8, IL-1β, IL-6, TNF-α and IL-10) by the LPS-stimulated neutrophils and PBMCs was determined using the commercial sandwich ELISA kits following the instructions (BD Biosciences, San Jose, CA, USA). The detection ranges were 3.1–100 pg/mL, 3.9–125 pg/mL, 4.7–150 pg/mL, 7.8–250 pg/mL, and 7.8–250 pg/mL for IL-8, IL-1β, IL-6, TNF-α and IL-10 ELISA kits, respectively. The secretion degree of a particular factor was calculated as a percentage compared to the control cell samples untreated by the tested compound (100% activity). VO was tested at 5–75 μM in both cellular models. Dexamethasone (DEX; 5–75 μM) and quercetin (QU; 5–75 μM) were used as positive controls. Before the experiments, all compounds were dissolved in water–DMSO mixtures and diluted with the culture medium to obtain the desired concentrations. The final DMSO levels in the reaction environment were at most 2.5% and were checked not to influence the results. A detailed description of the procedures is included in the Supplementary Materials. LPS (from *Escherichia coli* O111:B4) was purchased from Merck Millipore (Billerica, MA, USA), and all other reagents from Sigma-Aldrich (St. Louis, MO, USA). All measurements were performed in 96-well plates using a microplate reader (SYNERGY 4, BioTek, Winooski, VT, USA).

### 4.5. Evaluation of IL-6, LOX, NF-κB1, and NF-κB2 Gene Expression in PBMCs

#### 4.5.1. Cells Pretreatment

After the isolation of PBMCs from buffy coats, treatment with VO and stimulation with LPS, the plates were centrifuged (2000 rpm, 10 min, 25 °C), the supernatants were aspirated, and cells were washed with PBS. Next, the cells were suspended in RNAlater^®^ (Sigma-Aldrich, St. Louis, MO, USA) and stored according to the manufacturer’s instructions until RNA isolation.

#### 4.5.2. RNA Isolation

The total RNA was isolated from PBMCs exposed to VO at 5–75 μM final concentrations. Isolation was carried out using the mini-column method using the Total RNA Mini kit (A&A Biotechnology, Gdańsk, Poland). The concentration and purity of the obtained RNA were checked by spectrophotometric measurements. Based on the calculated ratios of the absorbance at 260 nm to the absorbance at 280 nm, only RNA samples with a ratio in the range of 1.8–2.0 were selected for further analysis.

#### 4.5.3. Reverse Transcription

The isolated RNA was used for a reverse transcription reaction to obtain cDNA. The reaction was performed using the High-Capacity cDNA Reverse Transcription Kit (Applied Biosystems, Waltham, MA, USA) according to the manufacturer’s protocol. The composition of the reaction mixture was selected so that the final RNA concentration in each sample was 0.005 µg/µL. The reaction temperature conditions and the volume of reagents for the reaction were used as recommended by the manufacturer.

#### 4.5.4. Qualitative PCR Analysis

To verify the presence of cDNA in the samples, a PCR reaction was performed using primers specific to the *GAPDH* reference gene sequence. Based on the electrophoretic separation of the PCR products, samples with a band of 96 bp in an agarose gel were selected for further analysis. The qualitative evaluation of the studied gene expression was carried out by amplifying the selected gene sequences using specific primers. The composition of the PCR reaction mixture for each sample included 5 µL of mix reagent, 0.70 µL of 10 µM each primer solution, 1 µL of cDNA template, and water to a final volume of 20 µL.

#### 4.5.5. Quantitative Analysis

Quantitative analysis of the relative expression level of *IL-6*, *LOX*, *NF-κB1*, and *NF-κB2* genes was carried out by the real-time PCR technique (RT-PCR) following the iTaq Universal SYBR Green Supermix reagent kit protocol (Bio-Rad, Hercules, CA, USA). Real-time amplification reactions were performed on a CFX Connect Real-Time PCR Detection System (Bio-Rad, Warszawa, Poland). The reaction mixture used to evaluate all analysed genes consisted of 5 µL of mix reagent, 0.25 µL of 10 µM each primer solution, 1 µL of cDNA template, and nuclease-free water to a final reaction volume of 10 µL. The sequences of the primers used for the reactions are listed in Table S1. For each sample, an amplification reaction was performed in 3 replications in separate wells on the plate, and for each experiment, the test and reference genes were amplified in parallel. In each measurement series, negative control without template cDNA was amplified. The temperature conditions for the RT-PCR reactions are presented in Table S2. Due to the use of a non-specific fluorescent dye in the reactions, melting curves for the amplification products were prepared after each reaction to determine its specificity. The mean of the obtained Ct values for *GAPDH* and tested genes was calculated. To assess the relative level of gene expression, the ΔΔ Ct method was used. The relative expression ratio (R), calculated as described previously [65], was expressed as a percentage of gene expression and analysed compared to the LPS-stimulated control without the investigated compound (taken as 100%). The results for the negative control (untreated with the investigated compound, non-stimulated cells) were included in the stimulated control and investigated compound results calculations.

### 4.6. Statistical Analyses

The results were expressed as the means ± standard deviation (SD) of replicate determinations. The data were tested for normality using the Shapiro–Wilk test, which indicated no significant deviation from a normal distribution (*p* > 0.05). For non-cellular anti-inflammatory activity tests, three independent experiments were performed. For cell-based models, three independent experiments were performed with cells isolated from five independent donors. The statistical analyses (calculation of SD, one-way analysis of variance, and HSD Tukey tests) were performed using Statistica Pl software version 13.3 for Windows (StatSoft Inc., Kraków, Poland), with *p*-values < 0.05 regarded as significant.

## 5. Conclusions

Our findings demonstrate for the first time that leaves of *G. procumbens* are an efficient plant source of VO. This megastigmane derivative is biocompatible with human cells and exhibits significant and dose-dependent antioxidant and anti-inflammatory activity in non-cellular in vitro tests and human immune cells ex vivo. Primarily, VO exerts promising potential as a direct inhibitor of the pro-inflammatory LOX enzyme, with the inhibitory activity comparable to that of the natural anti-inflammatory agent NDGA. Secondly, VO at 75 μM substantially reduces the ROS level generated during the oxidative burst of neutrophils by 54.8% compared to the *f*MLP-stimulated control. Thirdly, VO at the highest tested concentration significantly downregulates the release of pro-inflammatory cytokines (TNF-α) to 23.6% and 28.0% for neutrophils and PBMCs, respectively, and tissue-remodelling enzymes (ELA-2) by 51.3%, compared to the stimulated control in a model of LPS, *f*MLP or *f*MLP with cytochalasin B-stimulated human neutrophils and PBMCs, depending on the test. VO is also a potent inhibitor of *LOX*, *IL-6*, and *NF-κB1* gene expression in LPS-stimulated PBMCs. Incubation of the stimulated PBMCs with VO at concentrations of 50 μM and 75 μM led to a significant reduction in *LOX* gene expression to 18.2% and 12.3%, respectively, and *IL-6* gene expression to 45.5% and 31.1%, compared to the LPS-treated control cells. The observed effects support using VO as a promising non-cytotoxic natural modulator of pro-oxidant and pro-inflammatory functions of human immune cells, which might be applied in treating oxidative stress and inflammation-related disorders. However, since all present results are revealed in vitro and ex vivo, further studies are necessary to evaluate the potential toxicity and health-promoting effects of VO in vivo. The in-depth studies of mechanisms with a broader panel of potential targets and genes supplemented with expression studies at the protein level should also be the subject of further research.

## Figures and Tables

**Table 1:** Extraction yield and VO content in *G. procumbens* leaf, fruit and stem chloroform dry extracts (mg/g dw).

Compound/Extract	L.CHE	F.CHE	S.CHE
Extraction yield	75.70 ± 7.40 ^C^	46.47 ± 2.27 ^B^	39.92 ± 3.12 ^A^
VO ^a^	4.73 ± 0.35 ^B^	*n.d.*	1.30 ± 0.06 ^A^
VO ^b^	0.358 ± 0.026 ^B^	*n.d.*	0.052 ± 0.002 ^A^

Results are presented as means ± SD (*n* = 3). L.CHE: leaf chloroform dry extract; F.CHE: fruit chloroform dry extract; S.CHE: stem chloroform dry extract. Means with different superscript capital letters within the same row differ significantly (*p* < 0.05). Extraction yield calculated per dry weight (dw) of plant material. The content of (6*S*,9*R*)-vomifoliol (VO) was calculated per ^a^ dw of the extracts and a ^b^ dw of the plant material; *n.d.*—not detected [below limit of detection (LOD)].

**Table 2:** Inhibitory activity of VO towards pro-inflammatory enzymes.

Analyte	LOX	HYAL	COX-1	COX-2
	IC_50_ (mM)	IC_50_ (mM)	IC_50_ (mM)	IC_50_ (mM)
VO	0.54 ± 0.01 ^A^	0.85 ± 0.03 ^B^	>9.00	3.24 ± 0.16 ^C^
ASA	–	–	0.88 ± 0.03 ^A^	2.98 ± 0.01 ^B^
CX	–	–	–	0.027 ± 0.002 ^A^
NDGA	0.52 ± 0.02 ^A^	0.21 ± 0.01 ^A^	–	–

IC_50_: inhibition concentration in mM of the compound or positive control. VO: (6*S*,9*R*)-vomifoliol. The positive controls: ASA (acetylsalicylic acid), CX (celecoxib), and NDGA (nordihydroguaiaretic acid). Results are presented as mean values ± SD (*n* = 3). For each parameter, different superscript capital letters indicate significant differences (*p* < 0.05).

## Data Availability

The data presented in this study are available on request from the corresponding author.

## Supplementary Materials

The following supporting information can be downloaded at: https://www.mdpi.com/article/10.3390/ijms26041571/s1.

## Abbreviations

ASA	acetylsalicylic acid
BSTFA	*N*,*O*-bis(trimethylsilyl)trifluoroacetamide
COX	cyclooxygenase
CX	celecoxib
dw	dry weight
DEX	dexamethasone
ELA-2	elastase-2
FID	flame ionisation detector
*f*MLP	*N*-formyl-L-methionyl-L-leucyl-L-phenylalanine
GC	gas chromatography
HYAL	hyaluronidase
IL	interleukin
LPS	bacterial lipopolysaccharide from *Escherichia coli* O55:B5
LOX	lipoxygenase
MMP-9	matrix metalloproteinase-9
MS	mass spectrometry
NDGA	nordihydroguaiaretic acid
NF-κB	nuclear factor κ-light-chain-enhancer of activated B cells
NO	nitric oxide
PBMCs	human peripheral blood mononuclear cells
TMCS	trimethylchlorosilane
TNF-α	tumour necrosis factor-α
QU	quercetin
ROS	reactive oxygen species
SAAVNA	*N*-succinyl-alanine-alanine-valine-*p*-nitroanilide
STAT-3	signal transducer and activator of transcription 3
VO	(6*S*,9*R*)-6-hydroxy-3-oxo-α-ionol = (6*S*,9*R*)-vomifoliol = blumenol A
