# Supplementary material for: Anti-Inflammatory and Antioxidant Effects of (6S,9R)-Vomifoliol from Gaultheria procumbens L.: In Vitro and Ex Vivo Study in Human Immune Cell Models
Source: Int J Mol Sci. 2025 Feb 13;26(4):1571. doi: 10.3390/ijms26041571 (PMC11855001; doi:10.3390/ijms26041571)
Supplement: Supplementary file 1 [file ijms-26-01571-s001.zip › ijms-3428067-supplementary.pdf]

Article

Anti-Inflammatory and Antioxidant Effects of (6*S*,9*R*)-Vomifoliol from *Gaultheria procumbens* L.: In Vitro and Ex Vivo Study in Human Immune Cell Models

Piotr Michel [1,*], Anna Wajs-Bonikowska [2], Anna Magiera [1], Agnieszka Wosiak [3], Ewa Balcerczak [3], Monika Ewa Czerwińska [4,5] and Monika Anna Olszewska [1]

[1] Department of Pharmacognosy, Faculty of Pharmacy, Medical University of Lodz, Muszyńskiego 1, 90-151 Lodz, Poland; anna.magiera@umed.lodz.pl (A.M.); monika.olszewska@umed.lodz.pl (M.A.O.)
[2] Institute of Natural Products and Cosmetics, Faculty of Biotechnology and Food Sciences, Lodz University of Technology, Stefanowskiego 2/22, 90-537 Lodz, Poland; anna.wajs-bonikowska@p.lodz.pl
[3] Laboratory of Molecular Diagnostics and Pharmacogenomics, Department of Pharmaceutical Biochemistry and Molecular Diagnostics, Medical University of Lodz, Muszyńskiego 1, 90-151 Lodz, Poland; agnieszka.wosiak@umed.lodz.pl (A.W.); ewa.balcerczak@umed.lodz.pl (E.B.)
[4] Department of Biochemistry and Pharmacogenomics, Faculty of Pharmacy, Medical University of Warsaw, Banacha 1, 02-097 Warsaw, Poland; monika.czerwinska@wum.edu.pl
[5] Centre for Preclinical Research, Medical University of Warsaw, Banacha 1B, 02-097 Warsaw, Poland
* Correspondence: piotr.michel@umed.lodz.pl; Tel.: +48-426779169

Supplementary Materials

Lipoxygenase and Hyaluronidase Inhibition Test

Viability Assessment of Neutrophils and PBMCs

Evaluation of ROS Secretion by Neutrophils

Evaluation of IL-8, IL-1β, IL-6, IL-10, TNF-α, and MMP-9 Secretion by Human Immune Cells

Evaluation of ELA-2 Secretion by Neutrophils

References

Figure S1. Representative GC-FID-MS chromatograms of (A) wide (R_t: 25.00-85.50 min), and (B) narrow spectrum range (R_t: 34.00-48.00 min), of fruit (F.CHE), stem (S.CHE), and leaf (L.CHE) chloroform dry extracts with the (6*S*,9*R*)-vomifoliol (VO) peak marked (R_t: 39.96 ± 0.04 min).

Table S1. Sequences of the primers for PCR amplification.

Table S2. Time-temperature profile of PCR reaction.

Lipoxygenase and Hyaluronidase Inhibition Test

Inhibitory activity of the tested analytes (VO and reference compound) towards lipoxygenase was determined by a spectrophotometric method adjusted to a 96-well microtiter plates according to Matczak et al. [1]. Briefly, the assay began with the addition of 50 μL of the tested analyte solution prepared in sodium borate buffer (pH 9.0), followed by 50 μL of linoleic acid solution (134 μM) and 50 μL of lipoxygenase solution (167 U/mL) in sodium borate buffer (pH 9.0). The reagents were used in a volume ratio of 1:2:1 for working solutions of the tested analyte, linoleic acid, and enzyme, respectively. The reaction mixture was thoroughly shaken, and absorbance changes at 234 nm were recorded at 1-minute intervals over a 15-minute period. Activity of the tested analytes was calculated as inhibition percentage (% Inhibition) of lipoxygenase according to equation (S1):

$$\%_{\text{Inhibition}} = 100 \times \left(1 - \left(\frac{A_{AN} - A_{LA}}{A_{LOX}}\right)\right), \tag{S1}$$

where A_{AN} – absorbance of solution with the tested analyte, A_{LA} – absorbance of solution without the enzyme, A_{LOX} – absorbance of solution without the tested analyte (negative control). The absorbance value in the 10th minute of the reaction were used for the calculation.

Inhibitory activity of the tested analytes (VO and reference compound) towards hyaluronidase was determined by turbidimetric method adapted for 96-well microtiter plates as described previously [1]. Briefly, the assay commenced by adding 20 μL of the tested analyte solution in monosodium phosphate buffer (pH 7.0) to 40 μL of hyaluronidase solution (22.55 U/mL) in the same buffer. The mixture was then incubated in the dark at 37.0 ± 0.1°C for 10 minutes. Following this, 40 μL of hyaluronic acid solution (0.03%, w/v) in monosodium phosphate buffer (pH 5.35) was added, and the incubation was continued under the same conditions for an additional 45 minutes. Finally, 250 μL of bovine serum albumin solution (0.1%, w/v) in sodium acetate buffer (pH 3.75) was introduced, and the mixture was incubated at room temperature for 10 minutes. Changes in turbidity were then recorded at 600 nm. Activity of the tested analytes was calculated as inhibition percentage (% Inhibition) of hyaluronidase according to the equation (S2):

$$\%_{\text{Inhibition}} = 100 \times \left(1 - \left(\frac{A_{HA} - A_{AN}}{A_{HA} - A_{HYAL}}\right)\right), \tag{S2}$$

where A_{HA} – absorbance of solution without the enzyme, A_{HYAL} – absorbance of solution without the tested analyte (negative control), A_{AN} – absorbance of solution with the tested analyte.

Viability Assessment of Neutrophils and PBMCs

The potential cytotoxicity of VO was assessed by flow cytometry (BD FACSCalibur, BD Biosciences, San Jose, CA, USA) with propidium iodide (PI) staining and Triton X-100 solution as a positive control, according to Michel et al. [2] and Magiera et al. [3] for neutrophils and PBMCs, respectively. Prior to analysis, VO was dissolved in water-DMSO mixtures to prepare stock solutions, which were further diluted in RPMI 1640 culture medium to achieve the final working concentrations of 5-75 μM. The final DMSO levels in the reaction environment were at most 2.5%. Briefly, neutrophils and PBMCs (3.5×10^5 cells/mL) were cultured in 96-well plates in RPMI 1640 culture medium supplemented with 10% FBS, 10 mM HEPES, 1% penicillin-streptomycin, and 2 mM L-glutamine, in the presence or absence of VO. After incubation for 24 hours (for neutrophils) or 48 hours (for PBMCs), the cells were harvested, centrifuged (1500 RPM, 10 minutes, 4 °C), washed once with 500 μL of cold Ca^{2+}-free PBS, and centrifuged again. The supernatant was discarded, and the cell pellet was resuspended in 500 μL of PI solution (0.5 μg/mL), followed by

incubation at room temperature in the dark for 15 minutes. Flow cytometric analysis was then performed using a BD FACSCalibur system (BD Biosciences, San Jose, CA, USA), with 10,000 events recorded per sample. The proportion of PI-positive cells, indicating increased membrane permeability, was expressed as a percentage of PI(+) cells. Cells treated with Triton X-100 solution (98.6% and 99.3% of PI(+) cells) were used as positive controls in the neutrophil and PBMCs models, respectively.

Evaluation of ROS Secretion by Neutrophils

Reactive Oxygen Species (ROS) production in *f*MLP-stimulated neutrophils was assessed using a luminol-dependent chemiluminescence assay. Briefly, VO was dissolved in water-DMSO mixtures to prepare stock solutions, which were further diluted in HBSS culture medium to achieve the final working concentrations of 5-75 μM. The final DMSO levels in the reaction environment were at most 2.5%. After isolation, the neutrophils were suspended in HBSS. The assay was initiated by adding 50 μL of the tested compound solution to a 96-well plate, followed by 70 μL of the cell suspension (3.5×10^5 cells/mL), 50 μL of luminol (400 μg/mL), and finally, 30 μL of *f*MLP (9.9 μg/mL). Chemiluminescence was recorded every 2 minutes over a 40-minute period using a microplate reader (Synergy 4, BioTek, Winooski, VT, USA). ROS production was expressed as a percentage relative to the control (cells without the tested compound). Quercetin (QU) at 25-75 μM served as the positive control.

Evaluation of IL-8, IL-1β, IL-6, IL-10, TNF-α, and MMP-9 Secretion by Human Immune Cells

Neutrophils and PBMCs suspensions (940 μL; 3.5×10^5 cells/mL) in RPMI 1640 culture medium supplemented with 10% FBS, 10 mM HEPES, 1% penicillin-streptomycin, and 2 mM L-glutamine were pre-incubated in 96-well plates for 1 hour at 37 °C with 5% CO_2, in the presence or absence of the tested compound (50 μL). Prior to analysis, VO was dissolved in water-DMSO mixtures to prepare stock solutions, which were further diluted in RPMI 1640 culture medium to achieve the final working concentrations of 5-75 μM. The final DMSO levels in the reaction environment were at most 2.5%. The cell suspension was then stimulated with LPS (10 μL, 10 μg/mL) and incubated for 24 hours. Following incubation, the plates were centrifuged (2000 RPM, 10 minutes, 4 °C), and the supernatants were collected. Cytokine (IL-8, IL-1β, IL-6, IL-10, and TNF-α) and MMP-9 release from stimulated neutrophils and PBMCs was assessed using ELISA kits, following the manufacturer's instructions (BD Biosciences, San Jose, CA, USA; R&D Systems, Minneapolis, MN, USA), with absorbance measured using a microplate reader (Synergy 4, BioTek, Winooski, VT, USA). Cytokine production and enzyme release were expressed as a percentage relative to the control (cells without tested compound). Dexamethasone (DEX) at 25-75 μM served as the positive control.

Evaluation of ELA-2 Secretion by Neutrophils

The secretion of ELA-2 by *f*MLP-cytochalasin B-stimulated neutrophils was assessed using SAAVNA as a substrate. Neutrophil suspensions (200 μL; 4.0×10^6 cells/mL) in HBSS were pre-incubated in 96-well plates at 37 °C with 5% CO_2 for 15 minutes in the presence or absence of the tested compound (50 μL). Prior to analysis, VO was dissolved in water-DMSO mixtures to prepare stock solutions, which were further diluted in HBSS culture medium to achieve the final working concentrations of 5-75 μM. The final DMSO levels in the reaction environment were at most 2.5%. The cells were then stimulated with 50 μL of *f*MLP (5.6 μg/mL) and cytochalasin B (2.8 μg/mL) for 15 minutes. After incubation, the plates were placed on ice for 3 minutes and centrifuged (2000 RPM, 10 minutes, 4 °C).

The assay was conducted by transferring 100 μL of the freshly collected supernatants to a new 96-well plate, followed by the addition of 50 μL of SAAVNA solution (1.9

mg/mL). The release of *p*-nitrophenol was measured at 412 nm over a 300-minute period, with readings taken at 20-minute intervals, using a microplate reader (Synergy 4, BioTek, Winooski, VT, USA). The percentage of ELA-2 release was calculated relative to the control (cells without tested extracts). Quercetin (QU) at 25-75 μM served as the positive control.

References

1. Matczak, M.; Marchelak, A.; Michel, P.; Owczarek, A.; Piszczan, A.; Kolodziejczyk-Czepas, J.; Nowak, P.; Olszewska, M.A. *Sorbus domestica* L. leaf extracts as functional products: phytochemical profiling, cellular safety, pro-inflammatory enzymes inhibition and protective effects against oxidative stress *in vitro*. *J. Funct. Foods* **2018**, *40*, 207–218, doi:10.1016/j.jff.2017.10.046.
2. Michel, P.; Granica, S.; Magiera, A.; Rosińska, K.; Jurek, M.; Poraj, Ł.; Olszewska, M.A. Salicylate and procyanidin-rich stem extracts of *Gaultheria procumbens* L. inhibit pro-inflammatory enzymes and suppress pro-inflammatory and pro-oxidant functions of human neutrophils *ex vivo*. *Int. J. Mol. Sci.* **2019**, *20*, 1–17, doi:10.3390/ijms20071753.
3. Magiera, A.; Czerwińska, M.E.; Owczarek, A.; Marchelak, A.; Granica, S.; Olszewska, M.A. Polyphenol-enriched extracts of *Prunus spinosa* fruits: Anti-inflammatory and antioxidant effects in human immune cells *ex vivo* in relation to phytochemical profile. *Molecules* **2022**, *27*, 1691, doi:10.3390/molecules27051691.

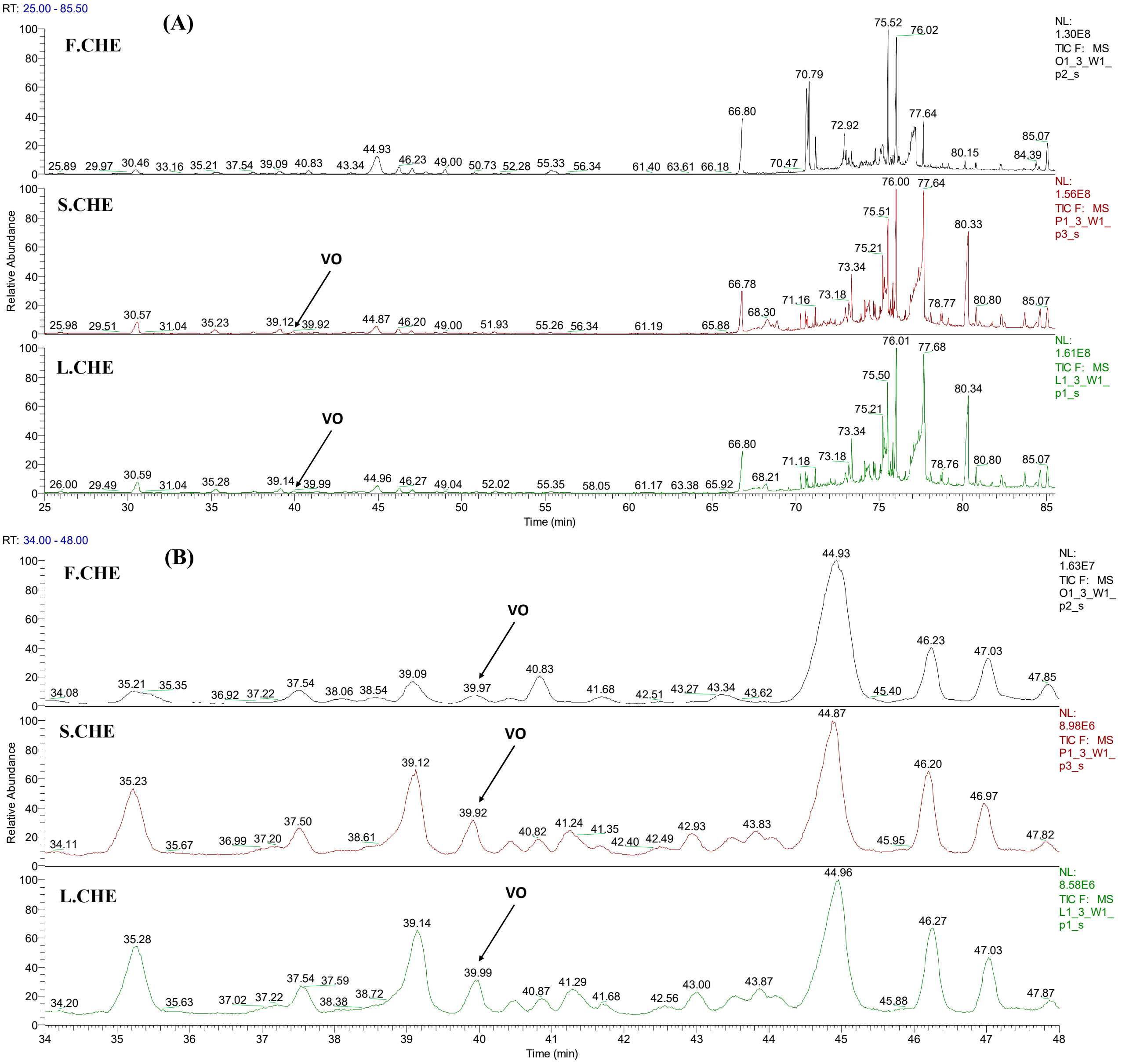

Figure S1. Representative GC-FID-MS chromatograms of (**A**) wide (R_t: 25.00-85.50 min), and (**B**) narrow spectrum range (R_t: 34.00-48.00 min), of fruit (F.CHE), stem (S.CHE), and leaf (L.CHE) chloroform dry extracts with the (6*S*,9*R*)-vomifoliol (VO) peak marked (R_t: 39.96 ± 0.04 min).

Table S1. Sequences of the primers for PCR amplification.

GAPDH Forward	5' ACA GTT GCC ATG TAG ACC 3'
GAPDH Reverse	5' TTG AGC ACA GGG TAC TTT A 3'
IL-6 Forward	5' GCA GAA AAA GGC AAA GAA TC 3'
IL-6 Reverse	5' CTA CAT TTG CCG AAG AGC 3'
LOX Forward	5' CAA CAT TAC CAC AGT ATG GAT G 3'
LOX Reverse	5' TAG TCA CAG GAT GTG TCT TC 3'
NF-κB1 Forward	5' CAC AAG GAG ACA TGA AAC AG 3'
NF-κB1 Reverse	5' CCC AGA GAC CTC ATA GTT G 3'
NF-κB2 Forward	5' CCA TGA CAG CAA ATC TCC 3'
NF-κB2 Reverse	5' TAA ACT TCA TCT CCA CCC C 3'

Table S2. Time-temperature profile of PCR reaction.

**PCR reaction step**		**Time**	**Temp [°C]**
initial denaturation		2 min	94.0
denaturation	× 34	15 sec	94.0
annealing		30 sec	64.0
elongation		1 min	68.0
finish elongation		5 min	68.0
